# Oral health-related quality of life and depressive symptoms in adults: longitudinal associations of the English Longitudinal Study of Ageing (ELSA)

**DOI:** 10.1186/s12903-023-03722-4

**Published:** 2023-12-20

**Authors:** Luisa Zwick, Norbert Schmitz, Mahdieh Shojaa

**Affiliations:** Department of Population-Based Medicine, University Hospital Tuebingen, Hoppe-Seyler-Str. 9, Tuebingen, 72076 Germany

**Keywords:** Oral health-related quality of life, Depression, Older adults

## Abstract

**Background:**

Little is known about the relationship between oral health status and depressive symptoms in adults in England. The aim of this study was to examine the longitudinal association between oral health parameters and depressive symptoms in adults in England.

**Methods:**

Data were obtained from the English Longitudinal Study of Aging (ELSA), which included information on self-rated oral health, oral impairment in daily life (Oral Impacts on Daily Performances, OIDP), and depressive symptoms (Center for Epidemiologic Studies Depression Scale, CES-D) in 6790 adults aged ≥ 50 years. Wave 3 data were used as baseline, while Waves 5 and 7 were used for follow-up assessments. Logistic regression was used to determine whether depressive symptoms at baseline anticipated self-rated oral health and OIDP and whether oral health status (at baseline) was associated with the development of depressive symptoms at follow-up assessment.

**Results:**

Participants with poor self-rated oral health were at higher risk of developing depressive symptoms, even after adjusting for behavioral, clinical, and sociodemographic characteristics (OR = 1.69, 95% CI 1.38–2.07). Similarly, having oral impacts on daily performances were associated with the development of depressive symptoms: The OR for developing depressive symptoms at Wave 5 or 7 was 2.19 (95% CI 1.62–2.96) after adjustment for all covariates. Participants with depressive symptoms at baseline were more likely to report poor self-rated oral health (OR = 1.93, 95% CI 1.52–2.44) or one or more oral impacts (OR = 1.86, 95% CI 1.45–2.40) at follow-up than those without depressive symptoms at baseline, even after adjusting for confounders.

**Conclusions:**

In the present study, a bidirectional association was found between depressive symptoms and poor oral health in older adults. Maintaining good oral health in older adults may be a protective factor against depressive symptoms. Therefore, more attention should be paid to promoting oral health awareness in older adults, including encouraging regular dental checkups, proper toothbrushing and flossing techniques, and healthy lifestyles.

## Background

Oral health is an increasingly important component of overall health, particularly for middle-aged and older adults. However, people of all ages suffer from a variety of oral health problems, including tooth loss, dental caries, periodontal disease, and temporomandibular disorders (TMD) [1]. Dental caries is the most prevalent oral disease, affecting 44% of the world's population [2]. According to the 2017 Global Disease Burden Study, oral diseases affected 3.5 billion people worldwide in 2016 [3].

Oral diseases also share risk factors with some noncommunicable diseases including cardiovascular disease, cancer, chronic respiratory disease, and diabetes such as a poor, sugary diet and tobacco and alcohol use [4].

Moreover, oral health problems are not always isolated from the rest of the body. On the contrary, they can have a direct impact on quality of life. Oral Health Related Quality of Life (OHRQoL) is a multidimensional construct that can include both professional measurement and subjective assessment of individual's oral health [5, 6].

Depression is one of the leading causes of disability [7] and the most common mental disorder, affecting approximately 300 million people worldwide [8]. One reason for the increased incidence of depression may be the aging population. It is expected that by 2030, one in six people will be 60 years or older [9].

Previous studies have found an association between OHRQoL and depression [10, 11]. Antidepressants can cause dry mouth and subsequent trouble swallowing, or bruxism [12]. Psychological reasons for poor oral health include lack of self-interest and self-care, which are common in people with depressive symptoms. This leads to the neglect of the dental hygiene, such as poor toothbrushing technique. Careless toothbrushing can lead to gum recession and exposed cervical, which in turn can lead to root caries [13].

On the other hand, an increasing number of lost and decayed teeth leads to problems with daily activities such as speaking and chewing. These limitations may lead to lower self-esteem, resulting in social distancing and poorer mental health [14].

Recent studies have found cross-sectional associations between oral health status and the occurrence of depressive symptoms [15, 16]. There is also evidence of increased risk of poor oral health in people with depression [11].

A Japanese longitudinal study showed that fewer teeth and oral health problems were associated with depression. Nevertheless, the study ran over a period of 3 years, while ours was conducted over 9 years [14]. However, longitudinal studies in this area are still rare. Therefore, the aim of this study was to assess the association between oral health status and depressive symptoms longitudinally. We also examined the role of some demographic, behavioural and clinical characteristics in the association between poor oral health status and depressive symptoms.

## Methods

### Study population

The current study used a combined methodological approach that included both cross-sectional and longitudinal designs. The data used in this study are from the English Longitudinal Study of Aging (ELSA), a continuous cohort study of adults living in private households in England and born before March 1, 1952. A total of 6790 adults were recruited, with a mean age (± SD) of 64 ± 9.5 years. ELSA collects data from the same respondents every two years, known as “Waves”. The first wave occurred in 2002, and the original sample was drawn from participants in the Health Survey for England (HSE) in 1998, 1999, and 2001. In Wave 3 (2006/2007), the sample was updated to retain the 50-to 53-year-old age group. Wave 3 data were used as baseline data in this study, while Wave 5 (2010/2011) and Wave 7 (2014/2015) were considered as follow-up assessments. Changes in both oral health parameters and depressive symptoms were compared across waves with baseline [17]. This study involved the analysis of a secondary data source. At the time of data collection ethical approval for all the ELSA waves was granted from the South Central – Berkshire Research Ethics Committee (21/SC/0030, 22nd March 2021). Informed consent was gained from all participants. All methods were performed in accordance with the relevant ELSA guidelines and regulations.

Of the 9771 participants in ELSA Wave 3, a total of 6790 individuals were included in the current study. Exclusion criteria were as follows: individuals younger than 50 years of age; participants with dementia and Alzheimer's disease; missing data in a given wave on depressive symptoms or oral health.

### Assessment of depression

Depressive symptoms during the past week were measured with the 8-item CES-D. This included two aspects of depression: depressed mood with five items (felt depressed, was happy, felt lonely, enjoyed life, and felt sad) and somatic complaints with three items (everything was an effort, restless sleep, and could not get going) [18]. Scores were categorized as no/low depressive symptoms (CES-D < 4) and elevated depressive symptoms (CES-D ≥ 4) according to previously published ELSA studies [19, 20]. While the original CES-D scale consists of 20 items, the 8-item CES-D is the most widely used version. Its reliability and validity are comparable to the 20-item CES-D, and its reduction to the most relevant questions makes it particularly appropriate for older adults [21]. Many studies have used the CES-D to assess depressive symptoms [22, 23].

### Assessment of oral health

Self-rated oral health information was collected by asking participants about their oral health status over the past year. Possible responses included "excellent", "very good", "good", "fair", and "poor". These were categorized as "good oral health" (excellent, very good, good) and "poor oral health" (fair and poor).

Oral health outcomes were assessed using the OIDP questionnaire. The OIDP is a self-administered instrument that measures the impact of oral conditions on an individual's ability to perform eight daily activities: eating and enjoying food; speaking and pronouncing clearly; cleaning teeth; sleeping and relaxing; smiling, laughing, and showing teeth without embarrassment; maintaining usual emotional state without being irritable; performing important work or social roles; and enjoying contact with people during the past six months [24]. Possible responses were categorized as "at least one oral condition" and "no oral condition". The dichotomization of the variables may affect the results. For example, if the only oral impairment is the inability to eat and enjoy eating, this may not be associated with poor oral health. However, the above categorizations are widely used in studies of self-rated oral health.

### Covariates

Several demographic, lifestyle, and clinical variables were included in the analysis of baseline characteristics. Sociodemographic variables included age, sex, and marital status (married, partnered, single, divorced, widowed, separated) dichotomized as married with a partner or single/ no partner. Socioeconomic status (SES) was estimated from the highest level of education (university degree or equivalent, less than a university degree, or no education). Lifestyle variables such as smoking status (current smoker or non-smoker) and physical activity (PA) level (sedentary/low, moderate, or high) were recorded. Frequency of alcohol consumption (a few times a year or never, once or twice a month, once or twice a week, three to four times a week, five to seven times a week), which was recoded into fewer categories (never/rare, occasional, 1–4 times/week, or ≥ 5 times/week), was also included in the analysis. In addition, existing comorbidities such as cardiovascular disease (arrhythmia, myocardial infarction, congestive heart failure, angina, heart murmur, and stroke) and diabetes were recorded.

### Statistical analysis

First, baseline characteristics and longitudinal associations between oral health and elevated depressive symptoms were calculated using descriptive statistics. Second, logistic regression analyses were conducted to assess the association between oral health at Wave 3 and the development of elevated depressive symptoms at Waves 5 and 7. Individuals with elevated depressive symptoms at Wave 3 were excluded from these analyses. Several models with different adjustments were considered: Model 1 assessed the strength of the association between oral health and depressive symptoms with adjustment for sociodemographic factors. Model 2 was adjusted for lifestyle factors; model 3 included clinical factors.

Third, logistic regression analyses were performed to assess the association between depressive symptoms at Wave 3 and the development of poor oral health at Waves 5 and 7. Individuals with poor oral health at Wave 3 were excluded and the same adjustment strategy as described above was applied.

As previous studies have used logistic regression to examine the association between oral health and depressive symptoms, this was considered appropriate for this study [10, 16].

Results are presented as odds ratios (OR) and confidence intervals (CI). Statistical analyses of the data were performed using IBM SPSS Statistics 28.0. Results with a *p*-value ≤ 0.05 were defined as statistically significant.

A test for multicollinearity was performed using Collinearity Diagnosis as part of logistic regression. All variables were tested and the highest variance inflation factor was 1.37, so all variables were considered to be independent contributors.

## Results

In the total sample, the mean age was 64 ± 9.5 years, with a range of 50 to 91 years. More than half of the participants (57.1%) were between 50 and 64 years old.

The majority of participants (56.2%) were female, and 56.5% were married or living together. In addition, 17.6% of participants reported poor oral health and 7.6% reported one or more oral conditions. Overall, 12.9% of participants reported elevated depressive symptoms.

Those who reported poor self-rated oral health at baseline had more depressive symptoms (23.7%) than those who reported good oral health (10.8%).

Individuals with elevated depressive symptoms were more likely to be older, female, less educated, a smoker, and less physically active than those without elevated depressive symptoms. A higher intake of alcohol consumption was associated with lower rates of depressive symptoms. Baseline characteristics of participants with respect to depression status are shown in Table 1.

**Table 1:** Baseline characteristics of the study sample regarding depressive symptoms; ELSA Wave 3 (*n* = 6790)

Variables	Total N (%)	No depressive symptoms (CES-D < 4) N (%)	Depressive symptoms (CES-D ≥ 4) N (%)	*P* values
**Age**				
50–64	3876 (57.1)	2455 (88.4)	322 (11.6)	** < 0.001**
65–74	1821 (26.8)	1501 (87.6)	213 (12.4)	
≥ 75	1093 (16.1)	861 (82.8)	179 (17.2)	
**Sex**				
Male	2977 (43.8)	2134 (90.8)	217 (9.2)	** < 0.001**
Female	3813 (56.2)	2683 (84.4)	497 (15.6)	
**Current marital status**				
Married/Partner	3833 (56.5)	2851 (91.1)	277 (8.9)	** < 0.001**
Single/Divorced/Widowed	2957 (43.5)	1966 (81.8)	437 (18.2)	
**Education**				
University degree or equivalent	1287 (19.0)	917 (94.1)	57 (5.9)	** < 0.001**
Less than university	2462 (36.4)	1808 (89.2)	220 (10.8)	
No professional qualification	3023 (44.6)	2092 (82.7)	437 (17.3)	
**Smoking status**				
Current smoker	976 (15.4)	554 (78.2)	154 (21.8)	** < 0.001**
Not current smoker	5362 (84.6)	4261 (88.4)	560 (11.6)	
**Frequency of alcohol consumption**				
Never/rarely	1455 (24.9)	1000 (81.0)	234 (19.0)	** < 0.001**
Occasional	726 (12.4)	556 (88.4)	73 (11.6)	
1–4 times/week	2282 (39.0)	1699 (91.0)	169 (9.0)	
≥ 5 times/week	1385 (23.7)	1046 (91.0)	104 (9.0)	
**Physical activity**				
Sedentary/low	1740 (25.7)	1118 (76.8)	337 (23.2)	** < 0.001**
Moderate	3574 (52.7)	2654 (89.7)	304 (10.3)	
High	1469 (21.7)	1040 (93.4)	73 (6.6)	
**CVD**				
Yes	1880 (27.7)	1363 (82.6)	287 (17.4)	** < 0.001**
No	4910 (72.3)	3454 (89.0)	427 (11.0)	
**Diabetes**				
Yes	583 (8.6)	412 (82.7)	86 (17.3)	**0.002**
No	6207 (91.4)	4405 (87.5)	628 (12.5)	
**Self-rated oral health**				
Good oral health	5596 (82.4)	4115 (89.2)	496 (10.8)	** < 0.001**
Poor oral health	1194 (17.6)	702 (76.3)	218 (23.7)	
**OIDP**				
No oral impacts	6203 (91.9)	4514 (88.6)	580 (11.4)	
≥ 1 oral impact	547 (8.1)	303 (69.3)	134 (30.7)	

*ELSA* English Longitudinal Study of Ageing, *CES-D* Center for Epidemiologic Studies Depression Scale, *CVD* Cardiovascular diseases including abnormal heart rhythm, myocardial infarction, congestive heart failure, angina, heart murmur, and stroke, Bold *p* values: *p* < 0.05

Participants with poor self-rated oral health (17.6%) or ≥ 1 oral impacts (8.1%) were less likely to be married and physically active, had a lower educational level, and were more likely to be smokers than those with good self-rated oral health/no oral impacts. Poor oral health was less common in individuals with higher alcohol intake. Results are shown in Table 2.

**Table 2:** Baseline characteristics of the study sample regarding self-rated oral health and OIDP; ELSA Wave 3 (*n* = 6790)

Variables	Total N (%)	Good self-rated oral health N (%)	Poor self-rated oral health N (%)	No oral impacts	≥ 1 oral impact
**Age**					
50–64	3876 (57.1)	3138 (81.0)	738 (19.0) *	3563 (92.4)	291 (7.6)
65–74	1821 (26.8)	1530 (84.0)	291 (16.0)	1649 (91.2)	159 (8.8)
≥ 75	1093 (16.1)	928 (84.9)	165 (15.1)	991 (91.1)	97 (8.9)
**Sex**					
Male	2977 (43.8)	2424 (81.4)	553 (18.6)	2722 (92.1)	233 (7.9)
Female	3813 (56.2)	3172 (83.2)	641 (16.8)	3481 (91.7)	314 (8.3)
**Current marital status**					
Married/Partner	3833 (56.5)	3243 (84.6)	590 (15.4) *	3530 (92.7)	276 (7.3) *
Single/Divorced/Widowed	2957 (43.5)	2353 (79.6)	604 (20.4)	2673 (90.8)	271 (9.2)
**Education**					
University degree or equivalent	1287 (19.0)	1102 (85.6)	185 (14.4) *	1203 (93.7)	81 (6.3) *
Less than university	2462 (36.4)	2055 (83.5)	407 (16.5)	2253 (92.0)	196 (8.0)
No professional qualification	3023 (44.6)	2423 (80.2)	600 (19.8)	2733 (91.1)	267 (8.9)
**Smoking status**					
Current smoker	976 (15.4)	710 (72.7)	266 (27.3) *	839 (86.2)	134 (13.8) *
Not current smoker	5362 (84.6)	4504 (84.0)	858 (16.0)	4944 (92.6)	393 (7.4)
**Frequency of alcohol consumption**					
Never/rarely	1455 (24.9)	1143 (78.6)	312 (21.4) *	1303 (89.6)	152 (10.4) *
Occasional	726 (12.4)	594 (81.8)	132 (18.2)	665 (91.6)	61 (8.4)
1–4 times/week	2282 (39.0)	1929 (84.5)	353 (15.5)	2143 (93.9)	139 (6.1)
≥ 5 times/week	1385 (23.7)	1202 (86.8)	183 (13.2)	1289 (93.1)	96 (6.9)
**Physical activity**					
Sedentary/low	1740 (25.7)	1344 (77.2)	396 (22.8) *	1511 (87.7)	211 (12.3) *
Moderate	3574 (52.7)	2960 (82.8)	614 (17.2)	3294 (92.6)	262 (7.4)
High	1469 (21.7)	1287 (87.6)	182 (12.4)	1392 (95.0)	74 (5.0)
**CVD**					
Yes	1880 (27.7)	1483 (78.9)	397 (21.1) *	1667 (89.3)	200 (10.7) *
No	4910 (72.3)	4113 (83.8)	797 (16.2)	4536 (92.9)	347 (7.1)
**Diabetes**					
Yes	583 (8.6)	446 (76.5)	137 (23.5) *	519 (90.3)	56 (9.7)
No	6207 (91.4)	5150 (83.0)	1057 (17.0)	5684 (92.0)	491 (8.0)
**Depressive symptoms**					
CES-D < 4	4817 (87.1)	4115 (85.4)	702 (14.6) *	4514 (93.7)	303 (6.3) *
CES-D ≥ 4	714 (12.9)	496 (59.5)	218 (30.5)	580 (81.2)	134 (18.8)

*ELSA* English Longitudinal Study of Ageing, *CES-D* Center for Epidemiologic Studies Depression Scale, *CVD* Cardiovascular diseases including abnormal heart rhythm, myocardial infarction, congestive heart failure, angina, heart murmur, and stroke

^*^: *p* value < 0.05

### The change in key variables

**Fig. 1:**
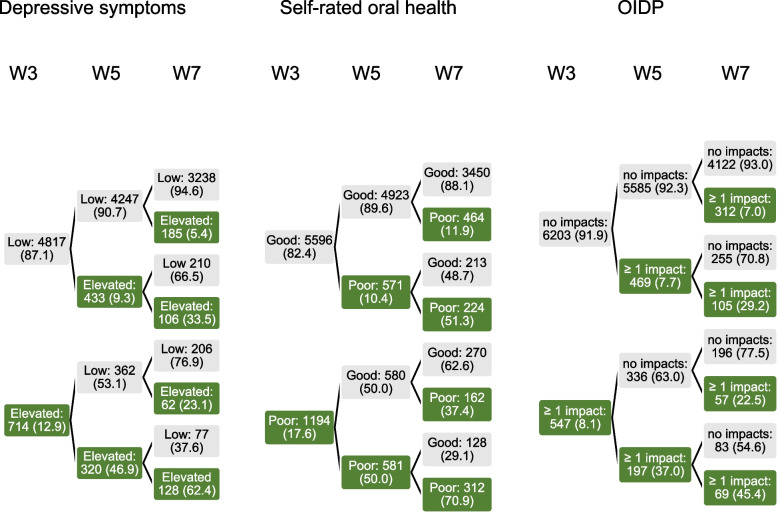
Development of depression, self-rated oral health and OIDP

As Fig. 1 shows, the majority of participants with low depressive symptoms at Wave 3 reported no change at Waves 5 and 7.

Neither self-rated oral health nor OIDP is a consistent variable, but the majority of participants with good oral health and no oral impairments maintained this condition throughout follow-up (Fig. 1).

### Longitudinal associations between oral health and depressive symptoms

Participants with elevated depressive symptoms who had good self-rated oral health at Wave 3 had worse self-rated oral health at Wave 5 (19.6%) than subjects with no or low depressive symptoms at Wave 3 (9.1%). Similarly, individuals with elevated depressive symptoms and no oral impairment at Wave 3 reported more oral impairment at Wave 5 (14.1%) compared to participants with no or low depressive symptoms at Wave 3 (7.0%).

Similarly, an association was found between poor oral health and the development of depressive symptoms. Specifically, the percentage of participants with poor self-rated oral health but no/low depressive symptoms at Wave 3 who developed elevated depressive symptoms at Wave 5 (15.6%) was higher than the percentage of participants with good self-rated oral health at Wave 3 (8.2%). Oral conditions also appeared to be associated with the development of depressive symptoms: 18.6% of participants with at least one oral condition but no depressive symptoms at Wave 3 reported depressive symptoms at Wave 5, whereas the percentage of depressed patients at Wave 5 who had no oral conditions at Wave 3 was 8.6%.

### Development of poor depressive symptoms

To better understand the longitudinal relationship between depressive symptoms and self-rated oral health while controlling for potential confounders, we constructed three logistic regression models. The results of the logistic regression analyses indicated that individuals with poor self-rated oral health at Wave 3 were 1.80 (95% CI: 1.46–2.22) times more likely to have depressive symptoms at Wave 5 and Wave 7. The odds ratio decreased only slightly in fully adjusted models (OR = 1.69, 95% CI: 1.38–2.07).

Slightly stronger associations were observed for oral health impairment: those with one or more oral impacts at Wave 3 were 2.36 (95% CI: 1.79–3.11) times more likely to have depressive symptoms at Wave 5 and Wave 7. The odds ratio decreased only slightly in fully adjusted models (OR = 2.19, 95% CI: 1.62–2.96). This may be attributed to the fact that depression can have many other causes, that were not included in the study. The results are presented in Table 3.

**Table 3:** Logistic regression analysis for the association of oral health at Wave 3 with depressive symptoms at Waves 5 and 7

	Model 1 OR (95% CI)	Model 2 OR (95% CI)	Model 3 OR (95% CI)
**Wave 5&7 depressive symptoms**			
W3 good self-rated oral health	1	1	1
W3 poor self-rated oral health	1.57 (1.31–1.88) 0.03	1.66 (1.46–2.02) 0.06	1.69 (1.38–2.07) 0.06
**Wave 5&7 depressive symptoms**			
W3 no oral impacts	1	1	1
W3 ≥ 1 oral impacts	2.34 (1.77–3.09) 0.03	2.18 (1.61–2.94) 0.06	2.19 (1.62–2.96) 0.06

Model 1: adjusted for sociodemographic variables

Model 2: Model 1 + lifestyle factors

Model 3: Model 2 + clinical factors

Participants with depressive symptoms in wave 3 were excluded

*OR* odds ratio, *CI* confidence interval, *W3* Wave 3

### Development of poor oral health

The results of the logistic regression analyses with poor self-rated oral health and one or more oral impacts are shown in Table 4, as the results at Waves 5 and 7 are presented in Table 4. Elevated depressive symptoms at Wave 3 were significantly associated with poor self-rated oral health at Waves 5 and 7 (OR: 1.94, 95% CI: 1.57–2.39). Similar results were found in the fully adjusted models (OR: 1.93, 95% CI: 1.52–2.44).

**Table 4:** Logistic regression analysis for associations of depressive symptoms at Wave 3 with oral health at Waves 5 and 7

	Model 1 OR (95% CI)	Model 2 OR (95% CI)	Model 3 OR (95% CI)
**Wave 5&7 self-rated oral health**			
W3 No depressive symptoms	1	1	1
W3 Depressive symptoms	1.91 (1.54–2.37) 0.02	1.93 (1.53–2.45) 0.04	1.93 (1.52–2.44) 0.04
**Wave 5&7 One or more oral impacts**			
W3 No depressive symptoms	1	1	1
W3 Depressive symptoms	1.99 (1.59–2.48) 0.02	1.88 (1.47–2.42) 0.03	1.86 (1.45–2.40) 0.03

Model 1: adjusted for sociodemographic variables

Model 2: Model 1 + lifestyle factors

Model 3: Model 2 + clinical factors

Participants with poor oral health in wave 3 were excluded

*OR* odds ratio, *CI* confidence interval, *W3* Wave 3

Similarly, the odds of having ≥ 1 oral impairment at Wave 5 and 7 were significantly higher among participants with elevated depressive symptoms at Wave 3 (OR:2.04, 95% CI: 1.64–2.54), even after adjustment for sociodemographic, lifestyle and clinical risk factors (OR:1.86, 95% CI: 1.45–2.40).

## Discussion

Using data from the English Longitudinal Study of Aging, a bidirectional association was found between depressive symptoms and poor oral health in older adults: Individuals with poor oral health and no depressive symptoms at baseline were more likely to report elevated depressive symptoms at 4- and 8-year follow-up than subjects with good oral health at baseline. In addition, individuals with depressive symptoms and good oral health at baseline were more likely to report poor oral health at 4- and 8-year follow-up than participants without depressive symptoms at baseline.

To our knowledge, this is the first study to examine the bidirectional association between depressive symptoms and oral health in a community sample of older adults over an eight-year period.

Our findings are consistent with previous cross-sectional studies examining the association between depressive symptoms and oral health [10, 15, 16, 25]. For instance, a study conducted in Germany with a sample of 3075 older adults reported an association between poorer oral health-related quality of life and higher risk of depression and anxiety [10].

Similarly, Aldosari et al. conducted a study of 9799 participants and found that depressive symptoms were associated with a higher number of missing teeth [16].

Some longitudinal studies also examined the association between depressive symptoms and self-rated oral health [5, 14, 26]: Ohi et al. found that impaired oral health-related quality of life predicted the development of depressive symptoms within 4 years in a sample of 296 older people [5]. Furthermore, in a study with a sample of 14279 elderly Japanese, depressive symptoms were more common in those with fewer teeth and more oral health problems [14]. What our study adds to these findings is the bidirectional approach and repeated assessment of depressive symptoms and oral health in a large, community-based sample.

The exact mechanisms behind the link between oral health and depression are not yet fully understood. However, it is likely that multiple factors are involved. For example, poor oral health can lead to pain and discomfort, which can affect quality of life and exacerbates depressive symptoms [25]. In addition, poor oral health can lead to social isolation and low self-esteem, which are known risk factors for depression [27]. Conversely, depression can have a negative impact on oral health, as people with depression are more likely to neglect their oral hygiene [15] and engage in unhealthy behaviors such as smoking, alcohol consumption [12], and reduced physical activity, which in turn can contribute to oral health problems [28]. Alcohol consumption has been associated with poor oral health [29], and depressive symptoms [30] in previous studies. However, in our study, higher alcohol consumption was associated with lower levels of depressive symptoms, and poor oral health, which may be due to the subjective measure.

Previous studies have shown that low socioeconomic status, as measured by income and education level, is associated with poorer oral health [31] and higher risk of depression [32]. High dental care costs and low level of education, which result in lower oral health awareness can lead to missed dental appointments. This can lead to the development of oral disease and a corresponding decline in overall health [33].

Moreover, depression and poor oral health share several common concomitants, including diabetes and cardiovascular disease (CVD). Depressive symptoms increase the risk of developing diabetes [34], which is strongly associated with the development of periodontitis [35]. Periodontitis has also been identified as a risk factor for CVD [36], which is often associated with depressive symptoms [37].

In addition, antidepressants can cause xerostomia and consequently reduce OHRQoL. Decreased salivary flow results in reduced buffering capacity for organic acids, leading to oral diseases such as dental caries [12]. Medication, especially for depression, was not included in the current study, which may have biased the results. However, dry mouth can lead to dental caries, especially in combination with a poor diet. This shows that many factors influence (oral) health, and it remains difficult to take them all into account.

The longitudinal design, large sample size, assessment of bivariate associations, and inclusion of potentially confounding variables can be considered strengths of the current study.

The present study also has some limitations. Depressive symptoms were measured using a self-report scale rather than clinical measures. The CES-D does not take into account the diagnosis, history, and treatment of depression. A structured clinical interview for depression would provide more information for the diagnosis of depression, which unfortunately was not available in ELSA. Oral health status was also assessed using a self-report scale, which may introduce reporting bias. ELSA is designed as a nationally representative sample, but the majority of participants in the current study were caucasian, which affects generalizability to other ethnic groups. ELSA also includes the UK population, which may not be generalizable to adults in other countries.

Attrition is always a problem in large older cohorts such as ELSA. It is possible that participants with depression are more likely to drop out of the study, which could lead to an underestimation of underlying associations. In addition, as with all observational studies, there may be unmeasured or unknown predictors. The large time intervals between waves with available data on oral health and depressive symptoms can also be seen as a limitation.

The implications of the link between oral health and depression are important for both patients and health care providers. It is important that patients understand the importance of good oral health to promote overall well-being and prevent depression. This includes regular dental checkups, proper brushing and flossing techniques, and healthy lifestyle choices such as abstaining from tobacco and limiting alcohol consumption.

## Conclusions

A healthcare provider’s ability to recognize the bidirectional relationship between oral health and depression and to address both aspects of a patient's health in a holistic and integrated manner is crucial. By recognizing the bidirectional relationship between these two aspects of health, we can work to improve overall well-being and reduce the burden of depression on individuals and society as a whole.

## Data Availability

The datasets generated and analysed during the current study are not publicly available, as permission to share them has not been granted by the English Longitudinal Study of Ageing (ELSA), but are available from the corresponding author on reasonable request.

## Abbreviations

ELSA: English Longitudinal Study of Ageing
OIDP: Oral Impacts on Daily Performances
CES-D: Center for Epidemiologic Studies Depression Scale
TMD: Temporomandibular disorders
OHRQoL: Oral Health Related Quality of Life
WHO: World Health Organization
